# Electrical Stimulation of Human Adipose-Derived Mesenchymal Stem Cells on O_2_ Plasma-Treated ITO Glass Promotes Osteogenic Differentiation

**DOI:** 10.3390/ijms232012490

**Published:** 2022-10-18

**Authors:** Seungho Baek, Heekyung Park, Fatma Dilara Igci, Donghyun Lee

**Affiliations:** 1 PCL Inc., 128, Beobwon-ro, Songpa-gu, Seoul 08510, Korea; 2 Department of Biomedical Engineering, School of Integrative Engineering, Chung-Ang University, 221 Heukseok-Dong, Dongjak-gu, Seoul 06974, Korea

**Keywords:** electrical stimulation platform, osteogenic differentiation, O_2_ plasma, indium tin oxide glass, adipose-derived mesenchymal stem cells

## Abstract

Electrical signals represent an essential form of cellular communication. For decades, electrical stimulation has been used effectively in clinical practice to enhance bone healing. However, the detailed mechanisms between electrical stimulation and bone healing are not well understood. In addition, there have been many difficulties in setting up a stable and efficient electrical stimulation system within the in vitro environment. Therefore, various conductive materials and electrical stimulation methods have been tested to establish an effective electrical stimulation system. Through these systems, many studies have been conducted on the effects of electrical stimulation on bone healing and osteogenic differentiation. However, previous studies were limited by the use of opaque conductive materials that obscure the cells; fluorescent observations and staining are known to be two of the critical methods to confirm the states of the cells. Indium tin oxide (ITO) glass is known to have excellent transparency and conductivity, but it is challenging to cultivate cells due to low cell adhesion characteristics. Therefore, we used O_2_ plasma treatment to increase the hydrophilicity and wettability of ITO glass. This enhanced cell affinity to the glass, providing a stable surface for the cells to attach. Then, electrical stimulation was applied with an amplitude range of 10 to 200 µA at a frequency of 10 Hz. Our results demonstrated that the osteogenic differentiation efficiency was maximized under the amplitude conditions of 10 µA and 50 µA. Accordingly, the results of our study suggest the development of an excellent platform in the field of biological research as a good tool to elucidate various mechanisms of cell bioactivity under electrical conditions.

## 1. Introduction

The function of bone is to support the body and protect internal organs [1,2]. In order to perform this function, bones are built to be strong, but they can be fractured by external forces [3,4]. In most cases, fractures take weeks or months to heal, but sometimes much longer, and moreover, 5–10% of fractures do not heal spontaneously [5,6]. In addition to external trauma, many patients suffer from bone loss due to osteoporosis or complications of diseases [2,6,7,8,9,10].

In patients with fractures or bone loss, the affected bone or bones are unable to support the body; therefore, daily activities and behaviors become hindered [11,12]. After a fracture occurs, a nonunion of bone (permanent failure of fracture/broken bone healing process) can result from three primary mechanisms: through tissue damage around the fracture area [13], through disruption of the blood supply to the healing tissue [14], or through continuous movement in the fracture site [15]. To overcome these complications, several studies have been conducted to effectively enhance the efficiency of osteogenic differentiation and reconstruction at a cellular level [16,17,18,19,20,21]. In line with such efforts, our group has cultured MC3T3-E1 pre-osteoblasts on carbon felt and showed that electrical stimulation enhances the osteogenic differentiation efficiency of MC3T3-E1 cells. The study was conducted in a hope to be able to ustilize local electrical stimulation to enhace the regeneration efficiency of the damaged bone tissues. However, because of the opacity of carbon felt, there was a limit to the optical and fluorescence analysis of the cells [22]. Certain studies have focused on the effectiveness of the use of mesenchymal stem cells in such damage recoveries. In a study by Xu et al., growth factors SDF-1 and BMP-2 were encapsulated in double-layered microspheres, and this growth factor delivery system was shown to increase the efficiency of osteogenic differentiation on bone marrow-derived mesenchymal stem cells [23].

**Scheme 1:**
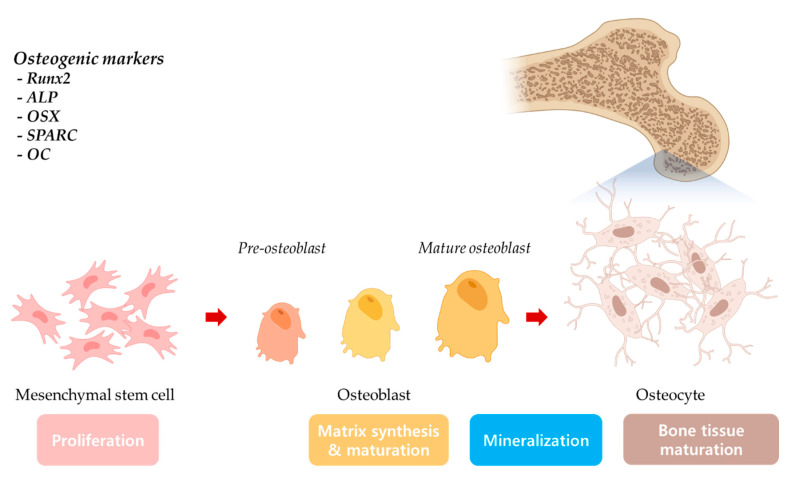
Mesenchymal stem cell osteogenic differentiation and osteogenic markers.

Mesenchymal stem cells have attracted close attention in the regenerative engineering field due to their multipotent properties [24]. Mesenchymal stem cells have been found to be able to be differentiated into adipogenic [25], chondrogenic [26], hepatogenic [27], neural [28], osteogenic (Scheme 1) [29], and tendon lineages [30]. These multipotent cells are obtained through isolation from various adult tissues, such as bone marrow [31], cord tissues [32], adipose tissue [33], skin [34], or brain [35]. Among the various sources, adipose-derived mesenchymal stem cells (ADMSCs) attracted researchers due to the ease of harvest from abundant adipose tissues [36,37].

Electrical stimulation has been employed in clinical application for more than decades to promote bone healing, mainly as a supplemental treatment for fracture treatments [38,39,40,41,42]. Although many studies have been conducted to explain the close interaction between electrical stimulation and osteogenic differentiation, the detailed mechanism of electrical stimulation that improves osteogenic differentiation has not yet been clearly explained [43]. One of the reasons lies in the selection of electrode materials; opaque electrodes cause difficulties in optical analysis [22,44]. Moreover, low cell affinity to the electrode surface also has been a hurdle for the establishment of a direct electrical stimulation system [45,46].

Many previous studies have demonstrated the bone healing efficacy of various types of electrical stimulation, including direct current (DC) [40], alternating current (AC) [47], combined magnetic fields (CMF) [48], and pulsed electromagnetic fields (PEMF) [49,50]. In this study, we have established an experimental system for electrical stimulation that can directly stimulate cells through indium tin oxide (ITO) glass. ITO glass was oxidized by O_2_ plasma treatment to enhance the cell attachment to the ITO electrode surface. O_2_ plasma exposure on the ITO glass surface has been shown to increase hydrophilicity, and, in turn, enhance cell attachment as well [51,52,53]. In the current study, we examined the effect of direct electrostimulation on osteogenic differentiation of human adipose-derived mesenchymal stem cells (hADMSCs) by evaluating alkaline phosphatase (ALP) activity, the degree of mineralization (alizarin red S analysis) and the expression of osteogenic-related genes. 

## 2. Results and Discussion

### 2.1. ITO Glass and O_2_ Plasma Treatment

**Figure 1:**
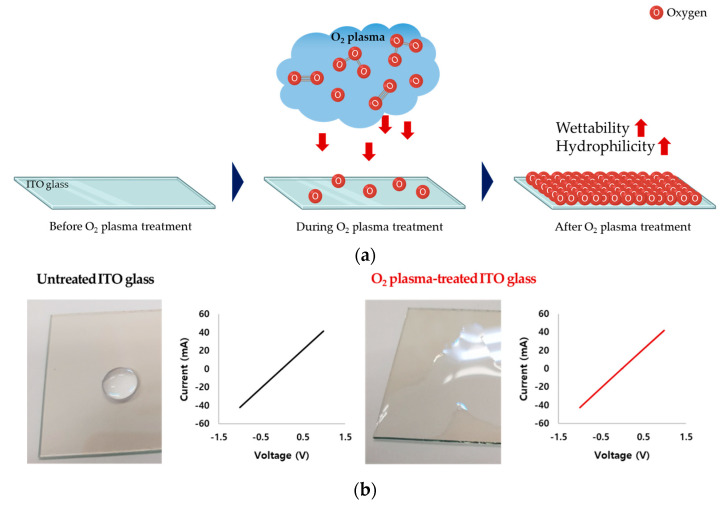

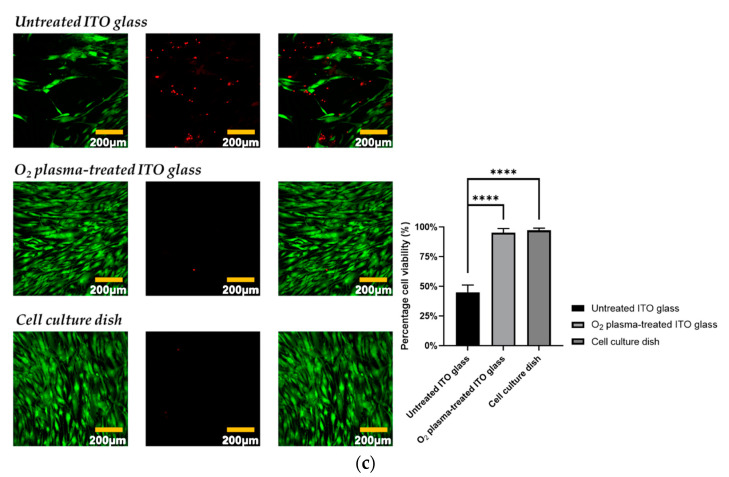
(**a**) Schematic of the O_2_ plasma treatment process for wettability and hydrophilicity of O_2_ glass surface. (**b**) Untreated ITO glass and O_2_ plasma-treated ITO glass conductivity test. (**c**) hADMSC cytotoxicity test on untreated ITO glass, O_2_ plasma-treated ITO glass, and a standard cell culture dish (green fluorescence—live cells, red fluorescence—dead cells)/a percentage of cell viability graph data were analyzed with one-way ANOVA and Tukey’s HSD post-hoc test (error bars represent the standard deviation (SD), **** *p* < 0.0001).

First, we applied O_2_ plasma treatment to increase the hydrophilicity and wettability of the ITO glass surface (Figure 1a). Increases in wettability and hydrophilicity were confirmed by optical microscopy, and conductivity was found not to be affected (Figure 1b). The cytotoxicity and cell attachment of the three conditions were compared: untreated ITO glass, O_2_ plasma-treated ITO glass, and cell culture dish. There was no significant difference in the viability of cells on the O_2_ plasma-treated ITO glass and those in the standard cell culture dish, whereas untreated ITO glass was cytotoxic, which can be due to low cell attachment (Figure 1c). These results confirmed that O_2_ plasma-treated ITO glass is suitable for our subsequent experiment of hADMSC differentiation with electrical stimulation.

### 2.2. Characterization of hADMSCs

**Figure 2:**
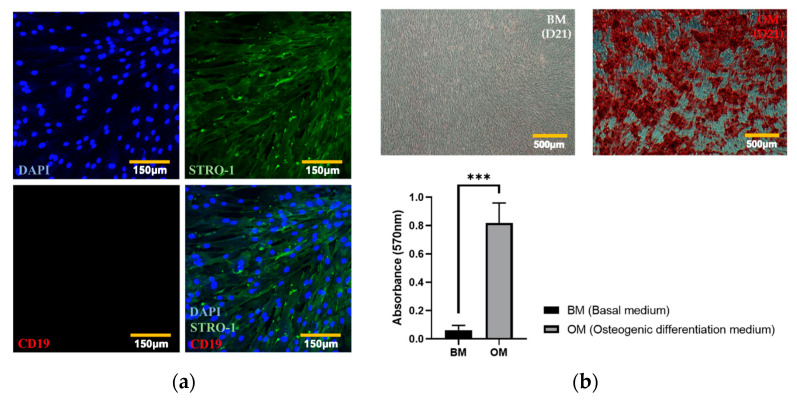
Characterization of mesenchymal stem cells. (**a**) Expressions of positive and negative markers were visualized using a confocal microscope, and the nuclei were counterstained with DAPI (STRO-1 (positive marker), CD19 (negative marker)). (**b**) ARS staining assay for mineralization quantified using the CPC method (*t*-test, *** *p* < 0.001).

To confirm that they exhibited typical characteristics of hADMSCs, we subjected our cells to IHC staining of positive and negative mesenchymal stem cell markers (STRO-1 and CD19, respectively [54,55]). As shown in Figure 2a, confocal microscopy (K1-Fluo; Nanoscope Systems, Daejeon, Korea) revealed that STRO-1, but not CD19, was expressed in the cells. To identify each cellular structure and to mark individual cells, the nuclei of hADMSCs were counterstained with DAPI. The ability of the hADMSCs to differentiate into osteocytes in the absence of electrical stimulation was then confirmed by an ARS staining assay and quantification by the CPC method. As shown in Figure 2b, hADMSCs cultured in OM were confirmed to induce bone differentiation, whereas hADMSCs cultured in BM did not differentiate, but only proliferated. The results of the ARS staining assay were quantified through the CPC method, and it was confirmed that the degree of mineralization in hADMSCs cultured with OM was significantly higher than that of hADMSCs cultured with BM. Therefore, the osteogenic differentiation ability of hADMSCs was demonstrated through ARS staining assay and CPC method and applied to our electrostimulation study.

### 2.3. hADMSC Cytotoxicity and Proliferation Assays after Electrical Stimulation and ALP Activity Assay

**Figure 3:**
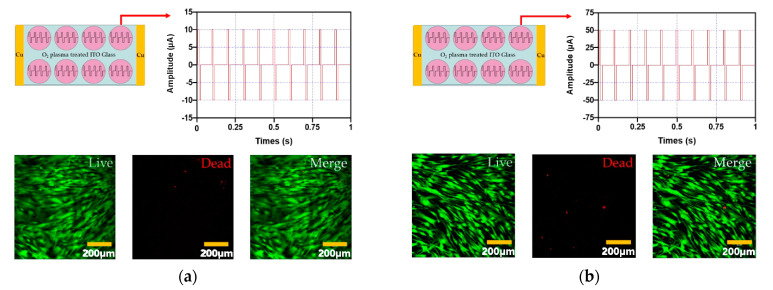

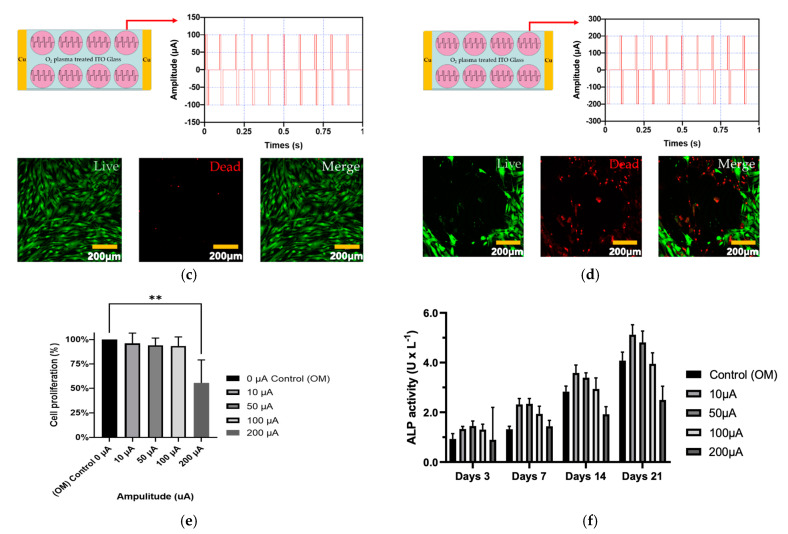
The cytotoxicity of electrical stimulation hADMSCs evaluated with the four different amplitude conditions (amplitude: (**a**) 10 µA, (**b**) 50 µA, (**c**) 100 µA, and (**d**) 200 µA and the frequency of all conditions was 10 Hz). In addition, the green fluorescence represents live cells and red fluorescence represents dead cells. (**e**) Cell proliferation of hADMSCs with electrical stimulation, (**f**) ALP activity assay of hADMSCs with electrical stimulation. These graph data were analyzed with one-way ANOVA (error bars represent the standard deviation (SD, ** *p* < 0.01). Finally, graph (**e**) was performed with Tukey’s HSD post-hoc test.

The cytotoxicity and proliferation of hADMSCs were studied on O_2_ plasma-treated ITO glass after electrical stimulation for 1 h (biphasic, 10 Hz) at four different amplitudes (10 µA, 50 µA, 100 µA, and 200 µA). Figure 3d shows that 200 µA electrical stimulation was more cytotoxic to hADMSCs than 10 µA, 50 µA, or 100 µA (Figure 3a–c). Further, the experimental groups that received 10 µA, 50 µA, or 100 µA electrical stimulation exhibited no change in proliferation relative to the control group, while the 200 µA group did exhibit a significant decrease in proliferation.

Next, the ALP activity of hADMSCs was assessed. As shown in Figure 3f, ALP activity was the highest after electrical stimulation of 10 µA (at 14 and 21 days), whereas, after electrical stimulation of 200 µA, no difference was observed relative to the control group (unstimulated). After 14 and 21 days, the group subjected to 200 µA electrical stimulation showed lower ALP activity than the control group. These findings confirmed that 10 µA electrical stimulation was optimal for inducing ALP activity (Figure 3f).

### 2.4. Analysis of Mineralization in ADMSCs Following Electrical Stimulation

**Figure 4:**
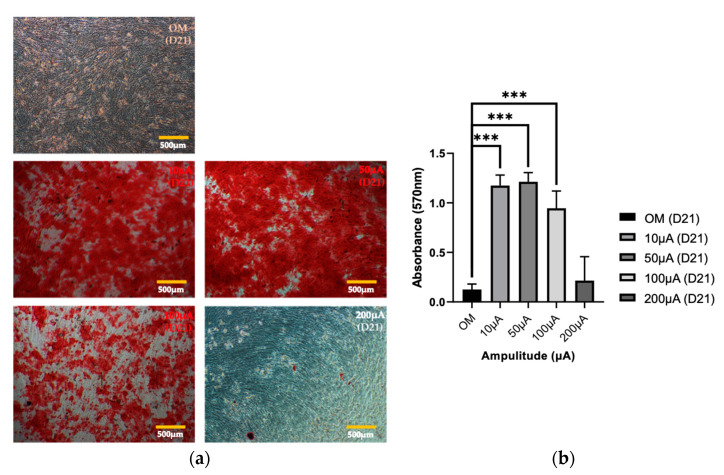
Mineralization in hADMSCs. (**a**) Optical images of ARS staining and (**b**) quantitative analysis using the CPC method (one-way ANOVA and Tukey’s HSD post-hoc test was performed, and error bars represent the standard deviation (SD), *** *p* < 0.001).

Next, we evaluated mineralization in hADMSCs after electrical stimulation, using an ARS staining assay and subsequent CPC-based quantification. The control and experimental groups were cultured in OM for 21 days, and then an ARS staining assay was performed. A high degree of mineralization was observed in the experimental groups that received 10 µA and 50 µA electrical stimulation, whereas there was no significant difference between the 200 µA group and the control group (Figure 4a,b). According to the results of the mineralization analysis, the experimental groups with significance compared to the control group were 10 µA, 20 µA, and 30 µA electrical stimuli groups, and the mineralization process hardly progressed under the 200 µA group.

### 2.5. Effect of Electrical Stimulation on the Expression of Osteogenic Genes

**Figure 5:**
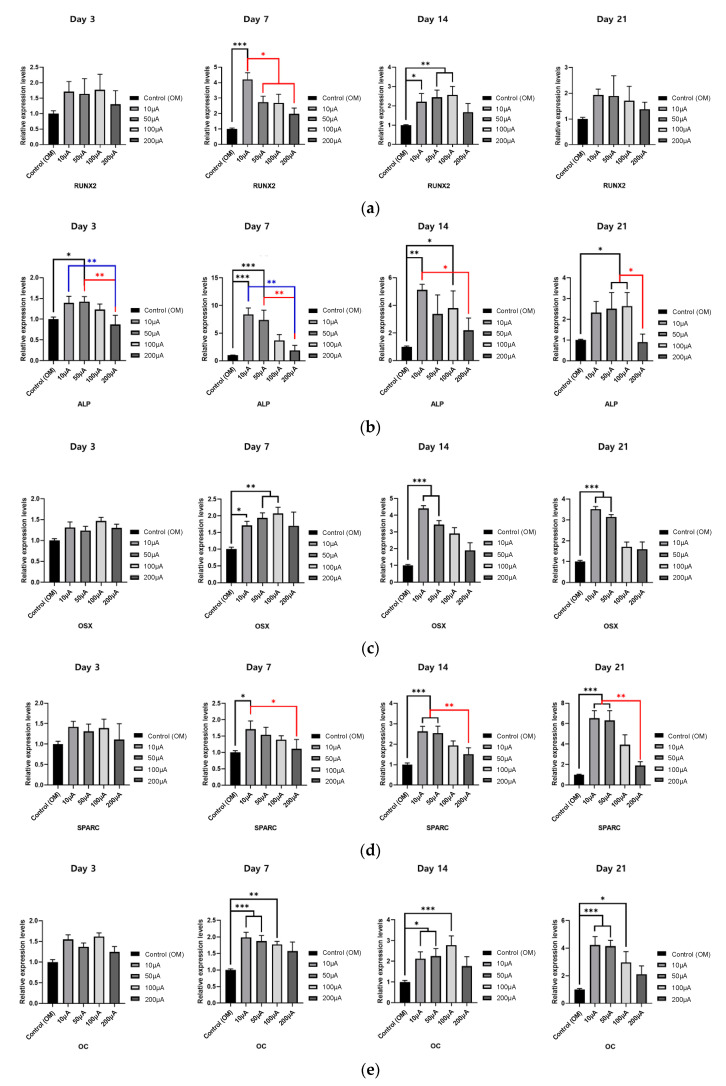
(**a**–**e**) The expression of five osteogenesis-related genes (*RUNX2*, *ALP*, *OSX*, *SPARC*, *OC*) in cells grown on O_2_ plasma-treated ITO glass with electrical stimulation (one-way ANOVA, Tukey’s HSD post-hoc test was performed, and error bars represent the standard deviation (SD), * *p* < 0.05, ** *p* < 0.01, *** *p* < 0.001). In all graphs, different line colors (red, blue, black lines) were used to show clear differences.

Next, to determine the effects of electrical stimulation on the osteogenic differentiation of hADMSCs, RT-qPCR was used to analyze the expression of the osteogenic genes *RUNX2*, *ALP*, *OSX*, *SPARC,* and *OC*. Electrical stimulation was applied from 3 to 21 days for one hour each day (Section 3.4). The expression of Runx2, a gene marker associated with the early- and mid-stage of osteogenic differentiation, was highest in the group that received 10 µA electrical stimulation, on day 7 (Figure 5a). Meanwhile, *ALP* was highest in the group that received 10 µA electrical stimulation, on day 7 and day 14 (Figure 5b). *OSX* gene expression was highest in the 100 µA electrical stimulation group on days 7 but in the 10 µA electrical stimulation group on days 14 and 21 (Figure 5c). Furthermore, on days 14 and 21, expression of the *SPARC* and *OC* genes, which encode proteins that affect mineralization, were expressed at significantly higher levels in the 10 µA and 50 µA electrical stimulation groups than they were in the control group. Electrical stimulation of 100 µA induced significant differences in gene expression compared with that of the control group for many of the conditions tested, but the expression levels of the genes were typically lower in the 100 µA group than they were in the 10 µA and 50 µA groups (Figure 5d,e). Similar to the cytotoxicity and ARS staining results (Figure 3 and Figure 4), 200 µA electrical stimulation did not induce a significant difference in the expression of any gene at any time point, compared to the control group (Figure 5). Taken together, our gene expression results show that the osteogenic differentiation of hADMSCs was significantly enhanced by 10–100 µA amplitude electrostimulation compared with the control group.

## 3. Methods and Materials 

### 3.1. Materials and Chemical Reagents

ITO was used as an electrode for direct electrical stimulation to hADMSCs and was purchased from BioScan Company (Seoul, Korea). A polydimethylsiloxane (PDMS) SYLGARD 184 silicone elastomer kit purchased from Dow Inc. (Cat. No. 31-00810-01, MI, USA), was used to fix cell chambers onto the ITO glass. The cell chambers were made by cutting the top of the 1000 µL pipette tip, purchased from Corning^®^ (Cat No. AX.T-1000-C, CA, USA). A potentiostat was purchased from AMETEK Scientific Instruments (VersaSTAT3, TN, USA) to control the electrical stimulation system. An O_2_ plama instrument was purchased from Femto Science Inc. (Gyeonggi-Do, Korea). A copper plate electrode purchased from Aron Science (Seoul, Korea) was used to connect the ITO glass with a potentiostat. Human adipose-derived mesenchymal stem cells (hADMSCs) were purchased from CEFO (Cat. No. CB-ADMSC-001, Seoul, Korea) for experiments. An alkaline phosphatase assay kit (ALP) was purchased from Abcam (Cambridge, UK) to analyze ALP activity. For cell culture, Dulbecco’s modified Eagle’s medium with low glucose, fetal bovine serum (FBS), and penicillin streptomycin solution (PS) were purchased from Thermo Fisher Scientific (Waltham, MA, USA). To evaluate the cell proliferation and cytotoxicity of the electrical stimulation, a Live/Dead Viability/Cytotoxicity kit for mammalian cells were purchased from Invitrogen (Carlsbad, CA, USA), and a Cell Proliferation Kit I (MTT) was purchased from Roche Life Sciences (Penzberg, Germany). To analyze gene expression, TRIzol^®^ was purchased from Thermo Fisher Scientific (Waltham, MA, USA) for RNA isolation, and a ReverTra Ace qPCR RT kit was purchased from Toyobo Co., Ltd (Cat No. FSQ-201, Osaka, Japan). for cDNA generation. All primary antibodies (STRO-1, ab214086, and CD19, ab134114), secondary antibodies (Goat Anti-Mouse IgG H&L (Alexa Fluor^®^ 488), ab150113 and Goat Anti-Rabbit IgG H&L (Alexa Fluor^®^ 647), ab150083) and DAPI staining solution (ab228549) used in immunohistochemistry experiments were purchased from Abcam (Cambridge, UK).

### 3.2. Experimental Setup of the Electrical Stimulation System

**Figure 6:**
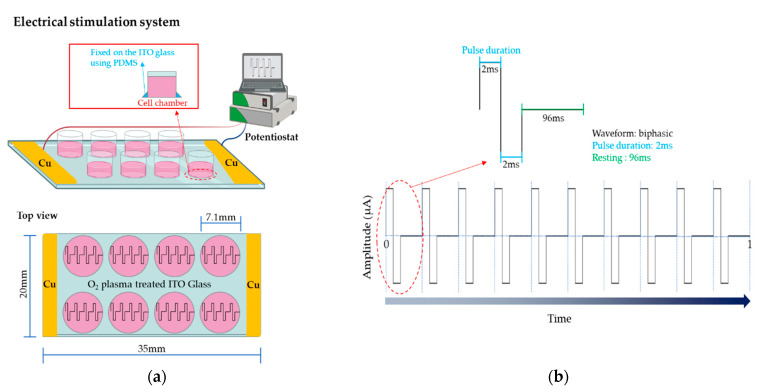
Schematic diagram of the electrical stimulation setup. (**a**) PDMS was used to fix the chambers onto the ITO glass. The yellow bars represent the Cu plate electrodes. (**b**) The electrical stimuli applied to adipose-derived mesenchymal stem cells in this study.

The electrical stimulation system employed in this study is shown in Figure 6a. Cu plate electrodes, connected to the potentiostat, were placed at each end of the ITO glass (Figure 6a), to enable a uniform current to flow through the ITO glass.

**Figure 7:**
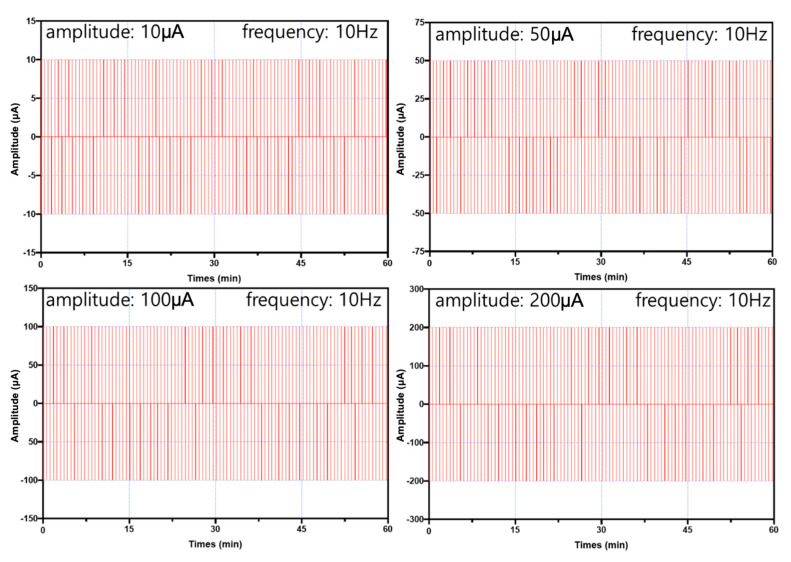
The four different electrical stimulation conditions (experimental group) employed in this study.

In this study, the electrical stimuli were applied as shown in Figure 6b. Four conditions of amplitude were considered as follows: 10 µA, 50 µA, 100 µA, or 200 µA (experimental group, Figure 7) and 0 µA (control). In addition, the frequency was set to 10 Hz in all conditions.

### 3.3. The O_2_ Plasma Treatment Process and Fixing the Cell Chamber

After the cell chambers were fixed onto the ITO glass, O_2_ plasma was generated to enhance the hydrophilicity and wettability of the ITO glass surface using an O_2_ plasma instrument for one minute. The operation conditions of the O_2_ plasma instrument were set to 100 W (power) and 20 kHz (frequency), respectively.

### 3.4. Cell Culture

**Figure 8:**
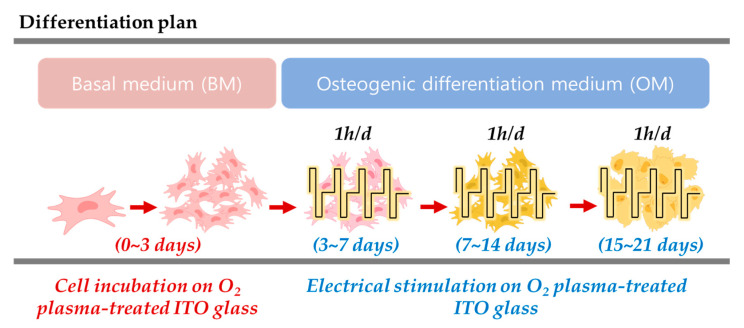
The osteogenic differentiation procedure under electrical stimulation.

To maintain self-renewing hADMSCs, cells were cultured in basal medium (BM). The cells were seeded at a density of 5000 cells/cm^2^. When the hADMSCs reached approximately 70–80% confluency, the cells were subcultured, and the medium was changed every 2–3 days. To electro stimuli the hADMSCs, the basal medium (BM) was replaced with osteogenic differentiation medium (OM), the supplements of which are shown in Table 1. The differentiation procedures are shown in Figure 8. The BM and OM contained 10% FBS and 1% PS, and the hADMSC incubation conditions were set to 37 °C and 5% CO_2_. All experiments were performed with cells at passage 4.

### 3.5. Immunohistochemistry (IHC)

Before the electrical stimulation experiments were initiated, IHC analysis was performed to confirm the identity of the hADMSCs. The cells were seeded at a density of 5000 cells/cm^2^, cultured in a 35 mm confocal glass bottom dish, and then were fixed by treatment with 4% paraformaldehyde in PBS (pH 7.4) for 10 min at room temperature. Then, fixed cells were treated to permeabilize with 0.05% Triton X-100 in PBS for 30 min, and then they were washed three times for 5 min. To prevent non-specific binding of the antibodies, the cells were incubated in a blocking solution of 1% bovine serum albumin (BSA) with 0.1% tween 20 in PBS (PBST) for 30 min. Then, blocked cells were incubated with diluted primary antibody (STRO-1 (1:500) and CD19 (1:500)) in 1% BSA, PBST at 4 °C overnight. Then, they were washed three times for 5 min (each in PBS). Fluorescent-conjugated secondary antibodies were diluted in 1% BSA, PBST for 1 h at room temperature. Then, the nuclei were stained with DAPI solution for 5 min, washed three times for 5 min (each in PBS), and filled with mounting solution. Finally, the stained cells were analyzed by confocal microscopy.

### 3.6. Cell Proliferation and Cytotoxicity Assay

The cytotoxicity and proliferation of the different groups of electrically stimulated hADMSCs were evaluated using a Live/Dead Viability/Cytotoxicity Kit for mammalian cells and a Cell Proliferation Kit I (MTT solution), respectively. Cytotoxicity on hADMSCs was tested after their exposure to electrical stimulation, the ITO glass surface, and the O_2_ plasma-treated ITO glass surface. The cytotoxicity results were obtained by using a confocal microscope to count the live cells (green) and dead cells (red). The percentages of cell viability were calculated from Equation (1) [56] and analyzed using ImageJ version 1.48 (National Institute of Mental Health, Bethesda, MD, USA).

Percentage of cell viability (%) = live cells (green)/live + dead cells (green + red) × 100%(1)

To evaluate cell proliferation, the absorbance of the MTT solution that reacted with the cells was measured at 590 nm using a microplate reader (Synergy H1; BioTek, Winooski, VT, USA).

### 3.7. Alkaline Phosphatase Activity Assay (ALP)

For ALP assays, ADMSCs were fixed with 4% paraformaldehyde in PBS (pH 7.4) for 15 min. Then, the cells were rinsed three times with PBS, and an ALP activity kit was used to assess ALP activity as follows. Cells in the electrical stimulation and control groups were lysed with lysis buffer (20 mM Tris-HCl (pH 7.5), 1% Triton X-100 and 150 mM NaCl) and then were stored at 37.5 °C for 30 min. ALP activity was measured at 405 nm using a microplate reader (Synergy H1; BioTek, Winooski, VT, USA).

### 3.8. Assessment of Mineralization Using Alizarin Red S (ARS) Staining and Cetylpyridinium Chloride (CPC) Quantitation

To determine the degree of mineralization in hADMSCs, ARS assays were performed. After the cell medium was removed, the cells were fixed with 4% paraformaldehyde in PBS (pH 7.4) for 30 min and then stained with 1% ARS solution (pH 4.2) for 30 min at room temperature on a shaker. Then, the cells were rinsed three times with double distilled water (ddH_2_O) and dried at room temperature. ARS staining was confirmed using an optical microscope. To quantify the degree of mineralization in ARS samples, 10% (v/w) cetylpyridiniym chloride (CPC) in sodium phosphate solution was added to the ARS samples, the samples were incubated for 5 min and absorbance was measured on a microplate reader at 570 nm.

### 3.9. RT-qPCR

TRIzol^®^ reagent was used to isolate total RNA from ADMSCs 3, 7, 14, and 21 days after inducing osteogenesis with electrical stimulation. RNA was quantified by measuring absorbance at 260 nm (Nanodrop^TM^ One UV-Vis spectrophotometer; Thermo Scientific, MA, USA). Then, cDNA was synthesized from total RNA using a ReverTra Ace qPCR RT kit. Real-time PCR was performed using SYBR^TM^ green PCR master Mix and analyzed on a StepOnePlus system (Applied Biosystems, Warrington, UK). *GAPDH* was selected as a housekeeping gene. All primer sequences are shown in Table 2. Relative expression levels are expressed using the 2^−ΔΔCt^ method.

### 3.10. Statistical Analysis

All statistical analyses were performed using GraphPad Prism 8 software (GraphPad Software, Inc., San Diego, CA, USA). All numerical data were derived by analyzing more than three replicates. The significance of statistical values was analyzed by *t*-tests and one-way ANOVA.

## 4. Conclusions

In summary, we developed a system that can directly electrostimulate cells through O_2_ plasma treatment on an ITO glass surface. From cell experiments, electrical stimulation under specific conditions showed good osteogenic differentiation efficacy. A plasma procedure can make the ITO glass surface suitable for cell experiments; it is a straightforward method with the advantage of a fast experimental stimulus process. O_2_ plasma treatment increased the hydrophilicity and wettability of the electrode surface, and thereby hADMSC affinity, for the ITO glass surface. The highest degree of mineralization was confirmed in the cells that received 10 µA and 50 µA electrical stimulation. In addition, ALP activity and RT-qPCR data confirmed that the 10 µA and 50 µA electrical stimulation groups exhibited the most efficient osteogenic differentiation characteristics. Due to the ITO electrode’s transparent nature, fluorescent and optical observations were also made available as well. As a result, we were able to successfully establish a stable system for studying the effect of electrical stimulation on osteogenic differentiation at a cellular level. Multiple analyses, including fluorescence and optical analysis, confirmed that this electrical stimulus successfully promotes osteogenic differentiation efficiency. Overall, our electrical stimulation system is expected to be useful for future research on various electrophysiological findings.

## Figures and Tables

**Table 1:** Supplements added to the osteogenic differentiation medium used in this study.

Products	Final Concentrations (in Medium ^1^)	Ref
Dexamethasone	0.1 µM	[18]
L-ascorbic acid	50 µM	[19]
β-glycerophosphate	10 mM	[20]

^1^ Dulbecco’s modified Eagle’s medium with low glucose, 10% FBS, and 1% PS.

**Table 2:** Sequences of the primers used in RT-qPCR analysis of the expression of osteogenic genes.

Gene	Forward Primer Sequence (5′–3′)	Reverse Primer Sequence (5′–3′)
GAPDH	AACATCATCCCTGCCTCTACTG	CCTGCTTCACCACCTTCTTGAT
Runx2	ACGAGGCAAGAGTTTCACCT	TCAAGCTTCTGTCTGTGCCT
ALP	TGAGAGTGACGAGAAAGCCA	TCCGTCACGTTGTTCCTGTT
OSX	ACTTTGCTCCCCTTAATCCAGC	TCTAGCTGCCCACTATTTCCCA
SPARC	AAGAAACTGTGGCAGAGGTGA	TGTTTGCAGTGGTGGTTCTG
OC	TGCAGCCTTTGTGTCCAA	TGAAAGCCGATGTGGTCA

GAPDH = glyceraldehyde 3-phosphate dehydrogenase, Runx2 = Runt-related transcription factor 2, ALP = Alkaline phosphatase, OSX = Osterix, SPARC = Secreted protein acidic and rich in cysteine, OC6 = Osteocalcin.

## Data Availability

No new data were created or analyzed in this study. Data sharing is not applicable to this article.

## Abbreviations

ALP	alkaline phosphatase
ARS	alizarin red staining
BM	basal medium
CPC	cetylpyridiniumchloride
GAPDH	glyceraldehyde 3-phosphate dehydrogenase
IHC	immunohistochemistry
ITO	indium tin oxide
OC	osteocalcin
OSX	osterix
OM	osteogenic differentiation medium
PDMS	polydimethylsiloxane
RUNX2	runt-related transcription factor 2
SPARC	secreted protein acidic and rich in cysteine

## References

[1] Rey C., Combes C., Drouet C., Glimcher M.J. (2009). Bone mineral: Update on chemical composition and structure. Osteoporos. Int..

[2] Einhorn T.A. (1998). The cell and molecular biology of fracture healing. Clin. Orthop. Relat. Res..

[3] Dimitriou R., Jones E., McGonagle D., Giannoudis P.V. (2011). Bone regeneration: Current concepts and future directions. BMC Med..

[4] Cho T.J., Gerstenfeld L.C., Einhorn T.A. (2002). Differential temporal expression of members of the transforming growth factor β superfamily during murine fracture healing. J. Bone Miner. Res..

[5] Holmes D. (2017). Non-union bone fracture: A quicker fix. Nature.

[6] Ferguson C., Alpern E., Miclau T., Helms J.A. (1999). Does adult fracture repair recapitulate embryonic skeletal formation?. Mech. Dev..

[7] Marina N., Gebhardt M., Teot L., Gorlick R. (2004). Biology and Therapeutic Advances for Pediatric Osteosarcoma. Oncologist.

[8] Giannoudis P.V., Atkins R. (2007). Management of long-bone non-unions. Injury.

[9] Ashman O., Phillips A. (2013). Treatment of non-unions with bone defects: Which option and why?. Injury.

[10] Mallick S., Beyene Z., Suman D.K., Madhual A., Singh B.N., Srivastava P. (2019). Strategies towards Orthopaedic Tissue Engineered Graft Generation: Current Scenario and Application. Biotechnol. Bioprocess Eng..

[11] Wang T., Zhang X., Bikle D.D. (2016). Osteogenic Differentiation of Periosteal Cells During Fracture Healing. J. Cell. Physiol..

[12] Bishop J.A., Palanca A.A., Bellino M.J., Lowenberg D.W. (2012). Assessment of Compromised Fracture Healing. J. Am. Acad. Orthop. Surg..

[13] Calori G.M., Giannoudis P.V. (2011). Enhancement of fracture healing with the diamond concept: The role of the biological chamber. Injury.

[14] Calori G.M., Albisetti W., Agus A., Iori S., Tagliabue L. (2007). Risk factors contributing to fracture non-unions. Injury.

[15] Hak D.J. (2011). Management of Aseptic Tibial Nonunion. J. Am. Acad. Orthop. Surg..

[16] Liu Z., Dong L., Wang L., Wang X., Cheng K., Luo Z., Weng W. (2017). Mediation of cellular osteogenic differentiation through daily stimulation time based on polypyrrole planar electrodes. Sci. Rep..

[17] Lee D.J., Tseng H.C., Wong S.W., Wang Z., Deng M., Ko C.-C. (2015). Dopaminergic effects on in vitro osteogenesis. Bone Res..

[18] Yamanouchi K., Gotoh Y., Nagayama M. (1997). Dexamethasone enhances differentiation of human osteoblastic cells in vitro. J. Bone Miner. Metab..

[19] Choi K.-M., Seo Y.-K., Yoon H.-H., Song K.-Y., Kwon S.-Y., Lee H.-S., Park J.-K. (2008). Effect of ascorbic acid on bone marrow-derived mesenchymal stem cell proliferation and differentiation. J. Biosci. Bioeng..

[20] Kyllönen L., Haimi S., Mannerström B., Huhtala H., Rajala K.M., Skottman H., Sándor G.K., Miettinen S. (2013). Effects of different serum conditions on osteogenic differentiation of human adipose stem cells in vitro. Stem Cell Res. Ther..

[21] Lee Y., Kim J., Koo J.H., Kim T.-H., Kim D.-H. (2017). Nanomaterials for bioelectronics and integrated medical systems. Korean J. Chem. Eng..

[22] Kim D., Bae M., Kim J., Yoon S., Lee D. (2016). Potentiostatically Stimulated Osteogenic Differentiation in a Mammalian Pre-Osteoblast Cell Line (MC3T3-E1). Sci. Adv. Mater..

[23] Xu C., Xu J., Xiao L., Li Z., Xiao Y., Dargusch M., Lei C., He Y., Ye Q. (2018). Double-layered microsphere based dual growth factor delivery system for guided bone regeneration. RSC Adv..

[24] Rosenbaum A.J., Grande D.A., Dines J.S. (2008). The use of mesenchymal stem cells in tissue engineering: A global assessment. Organogenesis.

[25] Sibov T.T., Severino P., Marti L.C., Pavon L.F., Oliveira D.M., Tobo P.R., Campos A.H., Paes A.T., Amaro E., Gamarra L.F. (2012). Mesenchymal stem cells from umbilical cord blood: Parameters for isolation, characterization and adipogenic differentiation. Cytotechnology.

[26] Kim Y.G., Park U., Park B.J., Kim K. (2019). Exosome-mediated Bidirectional Signaling between Mesenchymal Stem Cells and Chondrocytes for Enhanced Chondrogenesis. Biotechnol. Bioprocess Eng..

[27] Ayatollahi M., Soleimani M., Tabei S.Z., Salmani M.K. (2011). Hepatogenic differentiation of mesenchymal stem cells induced by insulin like growth factor-I. World J. Stem Cells.

[28] Scuteri A., Miloso M., Foudah D., Orciani M., Cavaletti G., Tredici G. (2011). Mesenchymal Stem Cells Neuronal Differentiation Ability: A Real Perspective for Nervous System Repair?. Curr. Stem Cell Res. Ther..

[29] Hanna H., Mir L.M., Andre F.M. (2018). In vitro osteoblastic differentiation of mesenchymal stem cells generates cell layers with distinct properties. Stem Cell Res. Ther..

[30] Chaudhury S. (2012). Mesenchymal stem cell applications to tendon healing. Muscle Ligaments Tendons J..

[31] Baghaei K., Hashemi S.M., Tokhanbigli S., Rad A.A., Assadzadeh-Aghdaei H., Sharifian A., Zali M.R. (2017). Isolation, differentiation, and characterization of mesenchymal stem cells from human bone marrow. Gastroenterol. Hepatol. Bed Bench.

[32] Nagamura-Inoue H.H.T. (2014). Umbilical cord-derived mesenchymal stem cells: Their advantages and potential clinical utility. World J. Stem Cells.

[33] Li C.Y., Wu X.-Y., Tong J.-B., Yang X.-X., Zhao J.-L., Zheng Q.-F., Zhao G.-B., Ma Z.-J. (2015). Comparative analysis of human mesenchymal stem cells from bone marrow and adipose tissue under xeno-free conditions for cell therapy. Stem Cell Res. Ther..

[34] Orciani M., Di Primio R. (2013). Skin-Derived Mesenchymal Stem Cells: Isolation, Culture, and Characterization. Methods Mol. Biol..

[35] Appaix F., Nissou M.F., van der Sanden B., Dreyfus M., Berger F., Issartel J.P., Wion D. (2014). Brain mesenchymal stem cells: The other stem cells of the brain?. World J. Stem Cells.

[36] Rad M.R., Bohloli M., Rahnama M.A., Anbarlou A., Nazeman P., Khojasteh A. (2017). Impact of Tissue Harvesting Sites on the Cellular Behaviors of Adipose-Derived Stem Cells: Implication for Bone Tissue Engineering. Stem Cells Int..

[37] Cho H., Kim H., Kim Y.G., Kim K. (2019). Recent Clinical Trials in Adipose-derived Stem Cell Mediated Osteoarthritis Treatment. Biotechnol. Bioprocess Eng..

[38] Bassett C.A.L., Becker R.O., Brighton C.T., LaVine L., Rowley B.A. (1974). Panel discussion: To what extent can electrical stimulation be used in the treatment of human disorders?. Ann. N. Y. Acad. Sci..

[39] Aleem I.S., Aleem I., Evaniew N., Busse J., Yaszemski M., Agarwal A., Einhorn T., Bhandari M. (2016). Efficacy of Electrical Stimulators for Bone Healing: A Meta-Analysis of Randomized Sham-Controlled Trials. Sci. Rep..

[40] Heppenstall R.B. (1983). Constant Direct-current Treatment for Established Nonunion of the Tibia. Clin. Orthop. Relat. Res..

[41] Bassett C.A., Mitchell S.N., Schink M.M. (1982). Treatment of therapeutically resistant non-unions with bone grafts and pulsing electromagnetic fields. J. Bone Jt. Surg..

[42] Steinberg M.E., Brighton C.T., Corces A., Hayken G.D., Steinberg D.R., Strafford B., Tooze S.E., Fallon M. (1989). Osteonecrosis of the femoral head. Results of core decompression and grafting with and without electrical stimulation. Clin. Orthop. Relat. Res..

[43] Griffin M., Bayat A. (2011). Electrical Stimulation in Bone Healing: Critical Analysis by Evaluating Levels of Evidence. Eplasty.

[44] Zhu S., Jing W., Hu X., Huang Z., Cai Q., Xiaoping Y., Yang X. (2017). Time-dependent effect of electrical stimulation on osteogenic differentiation of bone mesenchymal stromal cells cultured on conductive nanofibers. J. Biomed. Mater. Res. Part A.

[45] Mobini S., Leppik L., Thottakkattumana Parameswaran V., Barker J.H. (2017). In vitro effect of direct current electrical stimulation on rat mesenchymal stem cells. PeerJ.

[46] Wang Y., Cui H., Wu Z., Wu N., Wang Z., Chen X., Wei Y., Zhang P. (2016). Modulation of Osteogenesis in MC3T3-E1 Cells by Different Frequency Electrical Stimulation. PLoS ONE.

[47] Brighton C.T., Okereke E., Pollack S.R., Clark C.C. (1992). In vitro bone-cell response to a capacitively coupled electrical field. The role of field strength, pulse pattern, and duty cycle. Clin. Orthop. Relat. Res..

[48] Phillips M., Baumhauer J., Sprague S., Zoltan J., Bhandari M. (2016). Use of Combined Magnetic Field Treatment for Fracture Nonunion. J. Long-Term Eff. Med. Implants.

[49] Aaron R.K., McK Ciombor D., Simon B.J. (2004). Treatment of Nonunions With Electric and Electromagnetic Fields. Clin. Orthop. Relat. Res..

[50] Guerkov H.H., Lohmann C., Liu Y., Dean D., Simon B.J., Heckman J.D., Schwartz Z., Boyan B.D. (2001). Pulsed Electromagnetic Fields Increase Growth Factor Release by Nonunion Cells. Clin. Orthop. Relat. Res..

[51] Kim H., Lee J., Park Y., Park C. (2002). Surface Characterization of O_2_-Plasma-Treated Indium-Tin-Oxide (ITO) Anodes for Organic Light-Emitting-Device Applications. J. Korean Phys. Soc..

[52] Ramsey W.S., Hertl W., Nowlan E.D., Binkowski N.J. (1984). Surface treatments and cell attachment. In Vitro.

[53] Soliman I.E.-S., Metawa A.E.-S., Aboelnasr M.A.H., Eraba K.T. (2018). Surface treatment of sol-gel bioglass using dielectric barrier discharge plasma to enhance growth of hydroxyapatite. Korean J. Chem. Eng..

[54] Kolf C.M., Cho E., Tuan R.S. (2007). Mesenchymal stromal cells. Biology of adult mesenchymal stem cells: Regulation of niche, self-renewal and differentiation. Arthritis Res. Ther..

[55] Dominici M., Le Blanc K., Mueller I., Slaper-Cortenbach I., Marini F.C., Krause D.S., Deans R.J., Keating A., Prockop D.J., Horwitz E.M. (2006). Minimal criteria for defining multipotent mesenchymal stromal cells. The International Society for Cellular Therapy position statement. Cytotherapy.

[56] Singh N.S., Kulkarni H., Pradhan L., Bahadur D. (2013). A multifunctional biphasic suspension of mesoporous silica encapsulated with YVO_4_:Eu^3+^ and Fe_3_O_4_ nanoparticles: Synergistic effect towards cancer therapy and imaging. Nanotechnology.

